# Myeloid cells in alcoholic liver diseases: Mechanism and prospect

**DOI:** 10.3389/fimmu.2022.971346

**Published:** 2022-08-10

**Authors:** Wentao Xu, Miaomiao Wu, Bangjie Chen, Hua Wang

**Affiliations:** ^1^ Department of Oncology, The First Affiliated Hospital of Anhui Medical University, Hefei, China; ^2^ Inflammation and Immune Mediated Diseases Laboratory of Anhui Province, Hefei, China

**Keywords:** myeloid cell, immunity, alcoholic liver disease, cell-cell communication, single-cell ‘omics

## Abstract

Alcoholic liver disease (ALD) is a leading chronic liver disease in which immune cells play a vital role. Myeloid cells have been extensively studied in ALD, including granulocytes, macrophages, monocytes, and dendritic cells, which are involved in the occurrence and progression of steatosis, inflammation, fibrosis, and eventual cirrhosis. These cells can be popularly targeted and regulated by factors from different sources, including cytokines secreted by other cells, extracellular vesicles, and substances in serum—for example, infiltration of monocytes or neutrophils, activation of Kupffer cells, and polarization of macrophages. These processes can affect and change the function and phenotype of myeloid cells. Here we mainly review the key mediators that affect the infiltration and function of mainly myeloid cells in ALD as well as their regulatory mechanisms on target cells, which may provide novel immunotherapeutic approaches. The single-cell multimodal omics of myeloid cells is also discussed to help transform them into basic research or therapeutic strategy of ALD clinically.

## Introduction

Liver diseases are a serious global health burden, causing two million demises worldwide annually (1). There is a far-flung range of liver diseases, including diversiform chronic or acute events such as simplex steatosis, alcoholic liver disease (ALD), drug hepatitis and steatohepatitis, and so on. Chronic liver diseases diagnosed clinically mainly include non-alcoholic fatty liver disease (NAFLD) and ALD, with mild clinical symptoms (2). Nevertheless, dramatically increased alcohol intake in a short-term is associated with an acute inflammatory manifestation of alcoholic hepatitis (AH) in ALD. The persistence of chronic or acute liver lesions frequently leads to fibrosis, cirrhosis, and even hepatocellular carcinoma (HCC). Besides this, the mechanisms of these pathological processes involve the drive of *in situ* liver innate immune cells and the pro-inflammatory cascade activated in circulation (3). Under the influence of the nature or stage of the disease, the liver immune environment of patients is more intricate and untoward to decipher.

Myeloid cells are derived from myeloid progenitor cells which commonly exist in bone marrow (4). This lineage includes monocytes, granulocytes, erythrocytes, and platelets and serves as a master component of the innate immune system and the first block of defense against infection. According to our previous work, this review is principally focused on macrophages and neutrophils. Macrophages pertain to a multitudinous and heterogeneous population rooted in the transient but malleable monocyte precursors (5). In addition, there is the special resident Kupffer cells (KCs) in the liver, which is differentiated by the adhesion of blood monocytes to the hepatic sinusoid wall. Divertingly, neutrophilic percolation is also a sign of AH in patients with chronic alcoholism or in mice (6). The lethal effect of liver neutrophils may be achieved by the generation of reactive oxygen species (ROS) and vast inflammatory substances (7). Extensive mouse and human research have shown that neutrophils and macrophages exert a pivotal part in the foundation, development, and reversion of liver diseases, including in guiding tissue remodeling. With the progress of technology, more and more myeloid cell subtypes have been identified and located by single-cell multimodal omics (8–12). Each myeloid type acts as a unique individual participating in the immune response. Identifying and quantifying the existence of each myeloid subtype is momentous to comprehend the patterns in which different cell clusters are activated by certain pathogens and to promote the regression of immune stress.

## Alcoholic liver diseases

ALD are a concerning part of global chronic liver diseases and can be a consequence of the chronic abuse of alcohol (13). Normally, the broad spectrum of ALD includes simple steatosis, AH, fibrosis, cirrhosis, and HCC (14). Ample evidence is provided showing that the progress in ALD is chiefly related with the scale and sustainment of alcohol consumption. The inchoate pathophysiological response to chronic alcohol consumption is reflected in fat amassment in hepatocytes, which appears to be reversible (15). Nevertheless, when hepatocytes are damaged, the release of damage-associated molecular patterns tends to attract the infiltration of the surrounding immune cells toward it. Myeloid cells (especially macrophages and neutrophils) follow and promote liver inflammation as well as the incidence of alcoholic liver fibrosis and cirrhosis (16). Cirrhosis ratio was enriched in throng with added alcohol consumption and is higher in people with AH. About 3–12% of patients with AH progress to cirrhosis yearly (1). Data from 2009 to 2016 in the United States show that people in the 25–34 age group experienced the highest annual increase in liver cirrhosis-related mortality, driven entirely by ALD (17). Roughly 75% of ALD patients were currently diagnosed after decompensated cirrhosis, which disqualified them from the first-rank pharmaceutical treatment for alcohol use disorders (18). Emerging reports expounded multifarious omics and biomarkers for ALD diagnosis gradually (19, 20). Niu et al. established a machine learning model based on proteomics, constructed a diagnostic model superior to the existing clinical analysis, and discovered novel circulating protein markers on the basis of confirming the previous diagnostic markers, which provided potential protein targets with diagnostic, prognostic, and therapeutic value for ALD (21). Currently, the treatment of ALD still mainly relies on abstinence and nutritional support but also affected by the lack of advanced and effective therapeutic breakthroughs (22). Divertingly, based on the inherent ability of KCs to absorb most nanomaterials efficiently and non-specifically and the importance of KCs in the process of ALD, it may be possible to target myeloid cells, especially KCs, in the treatment of ALD by nano-drugs. Similarly, the therapeutic effect of liver macrophage-targeted nanoparticles in the NASH model has also been effectively confirmed recently (23).

## Myeloid cells

Myeloid cells have been diffusely discussed in liver diseases and are involved in the occurrence and development of ALD to varying degrees. The myeloid population is capable of recognizing, ingesting, and degrading cellular debris, foreign bodies, or pathogens as the first preventer to antagonize infection (24). Their studies in the liver tend to focus on macrophages, monocytes, granulocytes, and dendritic cells (DCs). Based on our forepassed research, this paper focuses on the mononuclear system, inherent KCs, and neutrophils, besides their mechanisms and frontier orientations in ALD and cirrhosis.

## Neutrophils

Neutrophils (the most enriched granulocytes) are first-line responders to inflammation and infection and can attack and eliminate invasive microbes by phagocytosis, which play a vital role in immune and inflammatory reaction (25). During liver infection and damage, neutrophils patrolling the hepatic sinusoids are rapidly recruited to the site of injury through diversified means, including phagocytosis, ROS generation, degranulation, cytokine and chemokine production, and neutrophil extracellular trap promotion to clear the pathogens and maintain tissue homeostasis (26).

There is growing evidence that the quantity of hepatic neutrophils is related to the ponderance of ALD (27, 28). CXCL1 is a key factor leading to neutrophil infiltration during alcoholic liver injury. It is eminently raised in the liver of patients with AH; besides this, its corresponding receptor CXCR2 is also highly expressed in neutrophils, which, in turn, induces neutrophil infiltration (29). Lipocalin (LCN2)—also called neutrophil gelatinase-associated lipocalin—is a secretory glycoprotein primarily localized to neutrophils, which participates in innate immunity. The expression of LCN2 is increased in humans and mice with alcoholic liver disease. More importantly, LCN2^-/-^ mice possessed lessened neutrophil permeation, liver injury, and hepatic steatosis in contrast to wild-type controls. Furthermore, antibody-mediated LCN2 blockade was also protective to confront with alcohol-induced liver injury, suggesting that LCN2 may be an underlying therapeutic target in ALD (30, 31).

Sirtuin1 (SIRT1), an NAD^+^-dependent histone deacetylase, is concerned with the regulation of senium. Neutrophilic SIRT1 expression is reduced in patients with acute alcohol consumption, and the deficiency of SIRT1 gene in myeloid cells accelerates liver injury and inflammation caused by ethanol and downregulates the neutrophil miR-223 level, leading to increased secretion of IL1β, TNFα, CXCL1, and ROS (32). Notably, miR-223 exerts a protective role in diversiform liver inflammatory diseases as one of the amplest miRNAs in neutrophils. Especially in ALD, miR-223 can reduce ROS production in neutrophils by directly inhibiting IL-6 expression and inhibiting the expression of phagocytosis oxidase (phox) p47phox (33). Interestingly, a recent study found that neutrophils can also interact with bile duct cells in patients with AH. Specifically, cell adhesion molecules on neutrophils bind to ITGB1 in cholangiocytes, triggering RAC1-induced JNK activation and resulting in c-JUN-mediated reduction of ITPR3 in cholangiocytes, thereby exacerbating bile in patients with alcoholic hepatitis siltation (34).

Axel Périanin et al. report that the reduced neutrophil peroxidase liberates, and bactericidal activity observed in patients with decompensated alcoholic cirrhosis is mainly correlated with impaired activation of AKT, p38-MAP-kinase and ERK1/2 signaling, NOX2 degradation, and lack of mTOR-dependent translation mechanisms on neutrophils (35). Although some studies have demonstrated the negative role of neutrophils in AH, many challenges remain in targeting neutrophils as therapeutic targets for ALD considering the complex functions and regulatory mechanisms of neutrophils.

## Macrophages

Hepatic macrophages are universally composed of tissue-resident macrophages and infiltrating macrophages (36, 37). Macrophages are usually present in the liver sinusoids, and when a liver infection occurs, macrophages infiltrate the liver in large numbers. Contraposing to capture hepatic macrophages, liver CD45^+^ cells were selected by fluorescence-activated cell sorting, in which F4/80^hi^CD11b^int^ cells were derived from resident KCs and macrophages, whereas F4/80^int^CD11b^hi^ cells were identified as infiltrating monocytes (38). Infiltrating monocytes give rise to two macrophage subtypes, including Ly6C^lo^-derived monocytes with anti-inflammatory and protective phenotypes and Ly6C^hi^-derived monocytes exhibiting pro-inflammatory and tissue-damaging phenotypes. The increase of Ly6C^lo^/Ly6C^hi^ cell ratio was considered to antagonize alcohol-induced liver injury in Gao-binge-fed mice (39). Macrophages are also plastic cells that can change their phenotype into pro-inflammatory cells (M1-like macrophages) or anti-inflammatory cells (M2-like macrophages) in response to a different signaling phage (40). Confrontationally, the IL10 released by M2 KCs promoted the death of M1 KCs and protected the liver injury of mice exposed to alcohol (41). Otherwise, the phagocytosis of macrophages, especially KCs, is a vital part of liver ecology *in situ*. A proximate study has found that the repair of phagocytosis of macrophages also contributes to resist alcoholic liver injury (42). Until now, numerous studies have increasingly shown the vital function of macrophages in hepatic steatosis, injury, inflammation, fibrosis, and cirrhosis.

Gyongyi Szabo et al. reported enhancive macrophages and an increased expression of chemokine receptors (CCR2 and CCR5) in the liver of patients with ALD, with corresponding increases in circulating chemokine CCL2 and CCL5 levels. Importantly, disposing with the dual CCR2/5 inhibitor cenicriviroc significantly reduced the increase in infiltrating macrophages and production of pro-inflammatory macrophages of the liver in alcohol-fed mice (43). In addition, studies have shown that the heat shock protein 96 (GP96) is extremely expressed in human and mouse ALD liver. Compared with WT mice, alcohol-fed GP96KO mice had significantly reduced extents of steatosis, serum endotoxins, and pro-inflammatory cytokines. *In vitro*, the pharmacological inhibition of GP96 or knockdown of small interfering RNA attenuated the inflammatory response of primary macrophages, suggesting that the targeted inhibition of GP96 may be a promising macrophage-based therapy for ALD (44).

Extracellular vesicles (EVs) may also mediate macrophage infiltration and activation during ALD—for example, it has been shown that ALD mice have increased total circulating EVs and that ALD-EVs can cause changes in hepatocyte function and a pro-inflammatory macrophage phenotype in naive recipient mice after injection by binding to Hsp90 (45). Furthermore, a study showed that alcohol treatment resulted in the production of mtdsRNA-rich exosomes in human and mouse hepatocytes, which were transported to adjacent KCs, resulting in TLR3 activation and IL-1β production by KCs. The subsequent activation of KCs further promoted the recruitment of γδ T cells and the level of IL-17A in the inchoate stages of ALD (46).

Our recent study found that TLR2 and TLR3 deficiency ameliorated and exacerbated alcoholic liver injury, respectively, and importantly, we found that gallocatechin gallate can directly interact with TLR2/3 in KCs to induce the production of IL-10 to regulate the progression of ALD through NF-κB signal, suggesting an innovative strategy for the treatment of ALD (47).

**Figure 1:**
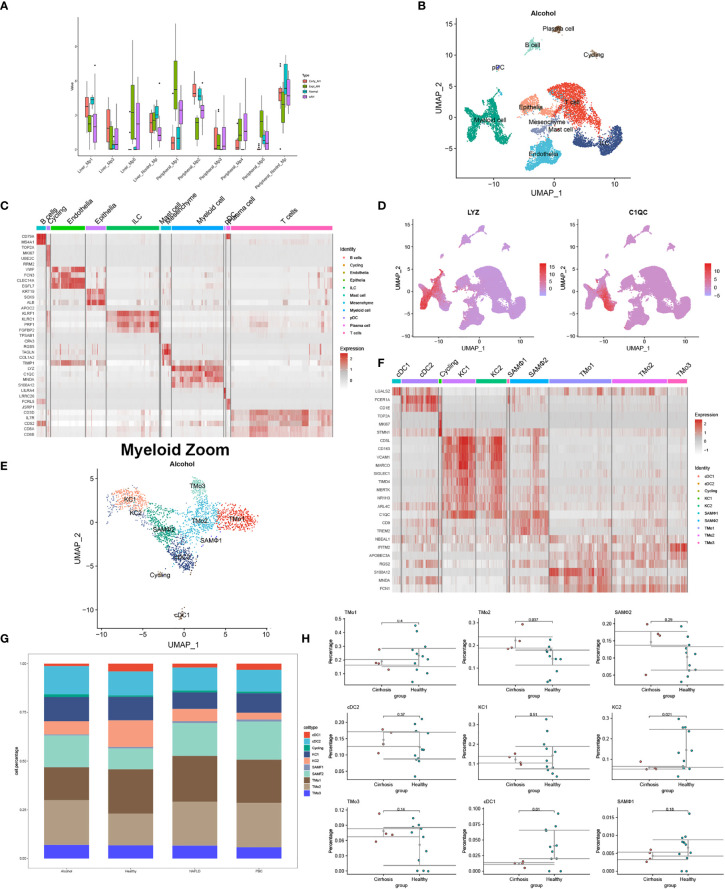
Identification of myeloid landscape in human alcoholic liver disease (ALD) and liver cirrhosis. **(A)** Changes of macrophages in the progression of ALD come from the mapping of single-cell sequencing data to RNA-seq. **(B)** Clustering 12382 cells from two cirrhotic human livers caused by alcohol and cell lineage as inferred from expression of marker gene signatures. ILC, innate lymphoid cell; pDC, plasmacytoid dendritic cell. **(C)** Heat map: cell types and marker genes. **(D)** Expression of LYZ and C1QC mRNA. **(E)** Myeloid cells were isolated from **(B)** and re-clustered with Seurat_4.1.0. **(F)** Heat map: myeloid cell types and marker genes. **(G)** The proportion of myeloid cells among normal patients and liver cirrhosis caused by alcohol, non-alcoholic fatty liver disease, and primary biliary cirrhosis. **(H)** Percentages of myeloid subpopulations in five healthy *versus* two cirrhotic livers, with a significant difference in the proportion of TMo2, scar-associated macrophages, KC2, and cDC1 between the two groups (*t*-test).

Moreover, according to single-cell sequencing and RNA-seq data from Adam Kim and Argemi et al., we recovered the changes of Mp1-Mp5 (LYZ+MARCO-) and non-inflammatory macrophages (MARCO+) in normal subjects, early alcohol-associated hepatitis, sAH with emergency liver transplants, and severe alcohol-associated hepatitis (8) (**Figure 1A**). Distinct macrophage subtypes from the liver *in situ* and in circulation have been described to perform different cell proportions with the evolution of ALD, indicating the research-based significance for disparate myeloid subtypes.

## Dendritic cells

DCs are capable of ingesting and processing antigens and expressing MHC molecules, which subsequently migrate to lymphoid organs to mediate the activation of naive T cells and secrete cytokines to initiate adaptive immunity and are powerful antigen-presenting cells (48). Classical DCs comprise two subtypes: type 1 cDCs (interacting with CD8+ T cells primarily *via* MHC-I) and type 2 cDCs (cDC2s) (presenting MHC-II-bound antigens to CD4+ T cells) (48). The function of DCs in hepatic steatosis, inflammation, and fibrosis remains controversial. In a thioacetamide- and leptin-induced liver fibrosis model, DCs activated hepatic stellate cells, NKT cells, and T cells by producing TNF-α, thus promoting the progression of liver fibrosis (36). However, although DCs promoted the mild activation of hepatic stellate cells, the depletion of DCs did not impact how liver fibrosis evolved in the carbon tetrachloride-induced liver fibrosis model (37).

The impact of alcohol intake on dendritic cell function has been of interest for nearly 20 years, but not much attention has been highlighted on the effect of DCs in the liver. In 2006, AH Lau et al. first reported that alcohol treatment impaired DC cell differentiation and function within the liver in *in vitro* experiments. However, *in vivo*, liver DCs were significantly less affected than spleen DCs after alcohol diet feeding in C57BL/6 mice (49). Earlier, Thomson et al. indicated that chronic ethanol intake impacts the *in vivo* migration of hepatic DCs to secondary lymphoid tissues (50). Comparatively interesting is a recent study by Alharshawi et al. They expounded that 12 weeks of alcohol consumption increased the hepatic plasmacytoid dendritic cells in female mice and that this increase was sex and organ specific. Furthermore, the mRNA expression level of CCR2 in the liver pDCs of female mice was significantly increased, and CCR2 controlled the pDC egress from the bone marrow at steady state and upon alcohol exposure, but not liver pDC recruitment (51). These studies suggest that DCs act as a momentous part in the progression of ALD and may be able to explain, to some extent, the population heterogeneity in the effect of alcohol intake on the liver.

## Vista

Although the subject of this review highlights myeloid cells, the liver serves as a complex multi-functional organ and constitutes a variety of cells from endoderm and mesoderm, including parenchyma cells and non-parenchyma cells. In disparate physiological and pathological conditions, including natural development, metabolism, aging, acute and chronic inflammation, scar formation, and other courses, myeloid cells showed different functions, accompanied by changes in cell interaction. The single co-culture experiment in liver research still has application defects in spatial and temporal backgrounds. With the progress of technology, organoids research is gradually emerging, which contains a variety of cell types to better simulate organ surroundings. Wang et al. established a novel model for the study of alcoholic liver disease through expandable hepatic organoids derived from human ESC, and Guan et al. used a human multi-lineage hepatic organoid model for the study of liver fibrosis (52, 53). Nevertheless, these studies were largely based on parenchymal cells, which would assuredly lead to a mature direction of organoid for myeloid cells in the liver.

Different from whole-tissue RNA sequencing analysis, scRNA-seq, spatial transcriptomics, and emerging single-cell multimodal omics endowed the study of transcriptional activity at the single-cell or spatial level, further broadening our understanding of tissue–cell interaction *in situ*—for example, under physiological conditions, scRNA-seq analyzed the livers of mice at 1, 3, 7, 21, and 56 days after birth and identified myeloid cell subsets at different time points, including a unique source of Dcn^+^ Mac from day 7 (11). At these periods, there was a stable Wnt signal in the interaction between KCs and hepatocytes, but a unique NOV-NOTCH1 signal interaction was produced in D7. We used GSE136103 (2 × NAFLD, 2 × ALD, and 1 × PBC) to reconstruct the single-cell landscape of two alcoholic liver cirrhosis samples and annotated the liver CD45- and CD45+ cells as well as the types of myeloid cells according to the method provided by P Ramachandran et al. (9) (**Figures 1B–H**). Prominent differences in myeloid cells such as tissue monocytes (TMo), scar-associated macrophages, KCs, and DCs were observed between cirrhotic patients and normal controls. Interestingly, alcoholic cirrhosis showed a different proportion of myeloid subsets from other disease states, showing more cDC2 and TMo3 and less TMo1 (**Figure 1G**). It presumed that myeloid subtypes may come into a different play, manifesting the value of exploration on myeloid subtypes in alcoholic liver cirrhosis or other liver diseases extended. Another scRNA-seq study found that the proportion and absolute number of Mp1–Mp5 and non-inflammatory macrophages in the peripheral blood of patients with sAH increased to varying degrees, although the article did not probe into the heterogeneity between these myeloid subtypes (8). Differently from the single cell study of alcoholic liver diseases, in livers of mice and human with NASH, researchers combined single-cell CITEseq, single-nuclei sequencing, spatial transcriptomics, and spatial proteomics to identify and locate lipid-associated macrophages derived from bile ducts and to determine the key axis in the development of KCs (10). Until now, there is no study pertaining to single-cell multimodal omics in ALD and cirrhosis ever yet, especially the role of rare myeloid-derived subtypes. This may be due to the limitations of technology and computing, the complexity of data integration, the high cost of application, and so on. The application of single-cell multi-group technology in more groups of layers requires methods that can integrate three or more types of data to effectively characterize the regulatory relationship between different groups of layers.

**Figure 2:**
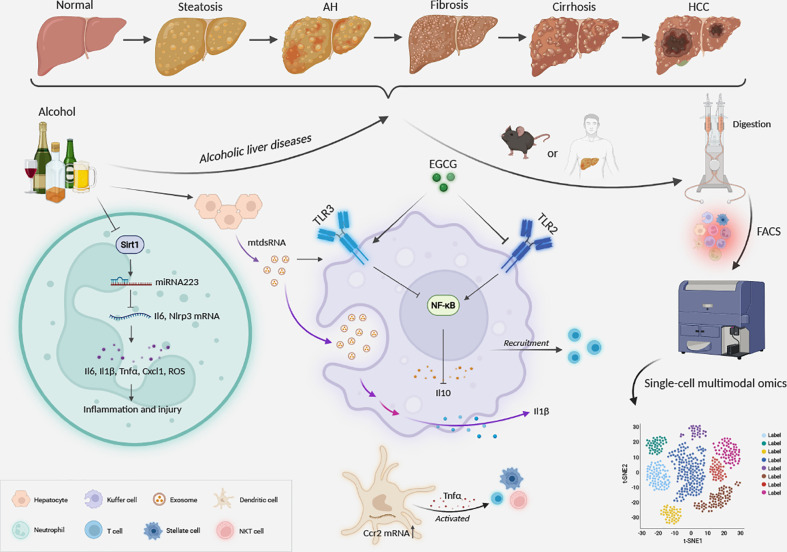
Mechanism and prospect of myeloid cells in alcoholic liver disease.

The study of myeloid subtypes possesses instructive significance in deciphering the involute immune environment and cellular interaction in the liver (**Figure 2**).

## Author contributions

HW and WX contributed to the work design, article construction and key revisions, and graphics design. WX, MW, and BC drafted the manuscript and reviewed the related articles. WX, MW, and BC contributed to analysis and final approval. All authors contributed to the article and approved the submitted version.

## Conflict of interest

The authors declare that the research was conducted in the absence of any commercial or financial relationships that could be construed as a potential conflict of interest.

## Publisher’s note

All claims expressed in this article are solely those of the authors and do not necessarily represent those of their affiliated organizations, or those of the publisher, the editors and the reviewers. Any product that may be evaluated in this article, or claim that may be made by its manufacturer, is not guaranteed or endorsed by the publisher.
